# Leveraging AI-driven nudge theory to enhance hand hygiene compliance: paving the path for future infection control

**DOI:** 10.3389/fpubh.2024.1522045

**Published:** 2025-01-23

**Authors:** Samiksha Bhattacharjee, Sudip Bhattacharya

**Affiliations:** ^1^Department of Clinical Pharmacology, All India Institute of Medical Sciences Deoghar (AIIMS Deoghar), Deoghar, India; ^2^Department of Community and Family Medicine, All India Institute of Medical Sciences Deoghar (AIIMS Deoghar), Deoghar, India

**Keywords:** hand hygiene, infection control, artificial intelligence, nudge theory, behavioral science

## Abstract

Hand hygiene is critical for preventing infections, yet maintaining compliance remains challenging across healthcare, schools, and communities. Despite strong evidence, lapses occur due to cognitive barriers, understaffing, limited resources, and antimicrobial resistance. Behavioral science highlights factors like time constraints and cognitive biases affecting adherence, with compliance rates as low as 40%. Nudge theory, developed by Thaler and Sunstein, offers promising solutions by using subtle interventions, like visual or auditory cues, to encourage hand hygiene without imposing strict regulations. Recent innovations integrate artificial intelligence (AI) with nudges, enhancing compliance through real-time feedback. AI-powered systems, such as smart dispensers and wearable devices, provide reminders using visual or auditory cues at critical moments. For example, dispensers may light up or chime when a healthcare worker enters a patient’s room, prompting hand hygiene. Studies show these AI-driven interventions significantly improve compliance, with rates increasing by up to 30% in some cases. AI can also analyze patterns of non-compliance, deploying personalized nudges during high-risk periods. Combining nudge theory with gamification, such as team-based competitions and rewards, further reinforces positive habits. However, implementing AI solutions in countries like India faces challenges, including limited resources, resistance to new technologies, and cultural barriers. Despite hurdles, integrating AI-driven nudges with behavioral strategies has the potential to transform hand hygiene practices. This approach fosters accountability, reduces infection rates, and ensures safer patient care by embedding compliance into daily routines, paving the way for sustainable improvements in infection control.

## Introduction

Hand hygiene is a key element in infection prevention, recognized worldwide for reducing pathogen transmission. Despite its effectiveness, adherence remains a challenge in healthcare, schools, and communities. A meta-analysis by Allegranzi and Pittet (1) highlighted that proper hand hygiene among healthcare workers significantly reduces healthcare-associated infections (HAIs), improving patient outcomes and lowering costs.

Despite strong evidence supporting the effectiveness of hand hygiene in preventing infections, maintaining compliance remains a persistent challenge. Infection control rates often fluctuate due to various interrelated factors, including lapses in hygiene practices, inadequate implementation of standardized protocols, and deficiencies in healthcare infrastructure. These challenges are exacerbated in resource-limited settings, where access to essential supplies such as soap, alcohol-based hand sanitizers, or even clean water may be inconsistent.

Understaffing in healthcare facilities significantly contributes to non-compliance, as healthcare workers often face overwhelming workloads and time constraints that deprioritize proper hand hygiene. Additionally, limited access to hygiene resources within high-pressure environments, such as emergency rooms or rural health centers, further hampers adherence to recommended practices. The situation is further complicated by the growing prevalence of antimicrobial-resistant (AMR) pathogens. AMR pathogens not only increase the risks associated with lapses in hygiene but also escalate the consequences of infection outbreaks. Poor hand hygiene provides a pathway for the transmission of these resistant strains, making the task of infection control even more critical and complex. These multifaceted challenges underscore the importance of not only promoting education and awareness around hand hygiene but also ensuring the availability of adequate resources, reinforcing protocols, and addressing systemic issues in healthcare infrastructure. Innovative solutions, such as the integration of technology and behavioral strategies, are increasingly being explored to overcome these barriers and ensure sustainable compliance with hand hygiene standards (1).

Are we looking in the wrong places? AI in healthcare could help tackle human behavior challenges. Despite education, healthcare workers face cognitive barriers to hand hygiene. AI and behavioral science, like nudge theory, offer solutions through real-time feedback, enhancing compliance and infection control.

## The role of human behavior in hand hygiene compliance

Healthcare workers (HCWs) navigate numerous cognitive demands daily, and hand hygiene frequently takes a backseat to more immediate tasks. Behavioral science identifies key factors that contribute to lapses in hygiene, including time constraints, risk perceptions, and cognitive biases (2). A systematic review by Eramus et al. points out the hand hygiene guideline compliance among 96 empirical studies were pretty low (40%) and a minority of those included studies only looked for behavioral determinants of hand hygiene stated previously. Nudge theory, developed by Richard Thaler and Cass Sunstein, suggests that small, subtle interventions, or “nudges,” can significantly influence behavior without limiting choices as humans are driven by “choice architecture” (3). Nudges are particularly useful in healthcare because they guide professionals toward the desired action—hand hygiene—without imposing stringent regulations (3).

A common example is the “default bias,” where individuals are more likely to follow preset guidelines if they do not require much effort to deviate from. It is a psychological tendency where individuals are more likely to stick with a preselected option or follow preset guidelines, primarily because it requires minimal effort to opt out or make an alternative choice. This bias leverages human preference for convenience and simplicity, capitalizing on the tendency to avoid additional cognitive or physical effort.

In the context of hand hygiene, default bias can be harnessed to encourage compliance by designing environments and systems that make the desired behavior—such as washing or sanitizing hands—the easiest or most natural action. For example, strategically placing hand sanitizer dispensers at prominent, unavoidable locations like the entrance of patient rooms or near medical equipment ensures that healthcare workers encounter these reminders as part of their routine workflow. The physical proximity and accessibility of these dispensers reduce the effort needed to perform hand hygiene, aligning with default bias.

Another application involves visual or auditory cues, such as dispensers that light up or emit a soft chime when approached. These cues serve as gentle nudges, reinforcing the preselected guideline of sanitizing hands before or after patient interaction. By incorporating these prompts into the environment, compliance becomes the path of least resistance, minimizing the likelihood of neglect. Default bias is particularly effective because it works subtly, without requiring conscious decision-making. By embedding the desired action into the default setting, healthcare institutions can promote consistent hand hygiene practices while reducing the cognitive burden on busy staff. This approach not only improves compliance but also fosters habitual behavior, contributing to better infection control outcomes. In hand hygiene, visual or auditory cues serve as nudges that subtly remind HCWs to wash their hands, even in low-risk settings where compliance is typically lower (4). By tapping into human psychology, nudges can trigger automatic compliance behaviors that would otherwise be neglected (4).

## AI-driven nudges can enhance hand hygiene practices

Recent innovations in AI are reshaping how healthcare settings implement nudges. Automated systems such as AI-driven smart dispensers and wearable devices and other automated electronic motoring system can monitor hand hygiene and offer real-time feedback based on individual behaviors (5). AI can go beyond passive monitoring and employ nudges like visual cues (lights on dispensers) or auditory prompts (alerts or chimes) to remind healthcare workers to wash their hands at the most opportune moments (5). For instance, when a healthcare worker approaches a patient room, the hand sanitizer dispenser could glow or emit a gentle chime as a nudge, reminding them to sanitize before entering. On these lights, a systematic review by Wang C et al. have found that, these can be a useful tool while keeping in mind the challenges that these bring along, such as issues of accuracy, usability, associated costs etc. (5).

Research shows that these AI-driven nudges have been highly effective in boosting compliance. A study involving AI-powered badges that provide auditory feedback every time a hand hygiene opportunity was missed showed a 30% increase in compliance rates (6). These interventions combine real-time feedback with gentle nudges, reinforcing habitual behavior without imposing direct control. It was found to be as effective as human observation in one of its kind trial done by Singh et al. (7).

Furthermore, AI-driven predictive analytics can identify patterns of non-compliance, allowing systems to deploy nudges at the most critical times. For example, AI can detect if a healthcare worker has a history of skipping hand hygiene during high-stress periods or after prolonged patient interaction. In such cases, personalized nudges, such as a voice prompt from the dispenser or a vibrating wearable, could pre-emptively remind the worker to wash their hands.

## Integrating nudge theory and AI for sustainable behavioral compliance

While technology plays a critical role, it is the combination of AI-driven nudges and behavioral feedback that can sustain long-term compliance. Nudge theory posits that consistent, subtle cues will eventually create habits that persist even when the prompts are no longer present (3–5). Healthcare settings that integrate nudges into their hand hygiene protocols use behavioral models, such as the cue-action-reward loop, to reinforce the desired action.

In hospitals, visual cues like color-changing lights and auditory signals such as chimes help reinforce hand hygiene, gradually becoming part of healthcare workers’ routines and reducing the effort needed to remember hygiene practices. Studies have shown that combining these nudges with gamification—another behavioral strategy—can enhance adherence (6). This technology can be leveraged here as when AI systems track team performance and offer rewards for high compliance, healthcare workers are more likely to sustain positive habits. One study at Netherlands involving nine public hospitals introduced team-based competitions with public recognition for high hand hygiene scores, leading to a significant (9%) improvement in compliance among nursing officers. Several institutions have successfully implemented AI-powered nudge systems to enhance hand hygiene compliance. At, New York City Health + Hospitals/South Brooklyn Health AHHR (automated hand hygiene reminder system) combined with real-time feedback increased hand hygiene compliance from around 86 to 89% in MSU wards over a 2 week period (8). The system was successfully implemented in that hospital and stands as a solid example to this initiative. Talking about India’s experiments, A Delhi based design studio “Quicksan” in partnership with Jhon’s Hopkin’s University launched a project called “Safehands” in labor rooms and district hospitals and community health centers across India aiming to find a solution to hand hygiene compliance. The device named was “HAIgeine,” consisting a camera to capture the hand movements, an AI based processing unit to give feedback. Although there was probably less flaw in the novel tool, rather the acceptance was something to be concerned for. There has been report that at some centers the physician’s particularly avoided the washbasin with this system enabled (9).

However, implementing AI-enabled nudge theory for hand hygiene in India faces challenges due to the country’s diverse healthcare system. In rural areas, inconsistent access to clean water and overstretched staff makes AI solutions difficult. Cultural shifts have complicated traditional hygiene practices, and resistance to AI systems is common due to unfamiliarity. High costs further limit adoption, especially in rural hospitals, widening healthcare inequalities. AI alone cannot address deeper behavioral issues like hierarchy, work pressure, and overconfidence.

The future of hand hygiene will combine AI with personalized nudges and wearable feedback, maintaining high compliance even during busy times (4, 5) AI can use hand hygiene data to deliver tailored nudges, such as personalized cues for specific situations like sanitizing after glove removal. This precision can significantly enhance compliance, lower infection rates, and improve patient outcomes.

## Conclusion

Combining AI-driven nudges with behavioral feedback effectively addresses hand hygiene compliance. This approach subtly influences behavior, boosts compliance, and fosters a culture of accountability, leading to safer patient care and better infection control.

## Recommendations

- To improve hand hygiene compliance, healthcare facilities should leverage behavioral insights like default bias and integrate them with technology-driven solutions. This can be achieved by designing workflows and environments where hand hygiene becomes the easiest and most natural action. For example, hand sanitizer dispensers should be strategically placed at critical points such as patient room entrances, ensuring their accessibility aligns with staff movements.
- Facilities should also adopt AI-enabled systems that provide real-time reminders through visual or auditory cues, such as lights or chimes, to prompt compliance. These systems should be tailored to individual behaviors, using predictive analytics to deploy nudges at high-risk times, such as during busy shifts or after prolonged patient interactions. Combining these interventions with gamification strategies, like team-based competitions and performance recognition, can further motivate healthcare workers to adhere to hygiene protocols.
- Additionally, efforts should focus on addressing systemic barriers, such as understaffing and resource limitations, to sustain compliance. This includes ensuring consistent availability of hygiene supplies, improving staff training, and fostering a culture of accountability through leadership support and ongoing feedback.
- For resource-limited settings, scalable and cost-effective solutions, like low-cost visual cues or simplified AI systems, should be prioritized. Implementing these measures will not only enhance hand hygiene practices but also contribute to reducing healthcare-associated infections and combating antimicrobial resistance, ultimately improving patient outcomes and safety.

## Data Availability

The original contributions presented in the study are included in the article/supplementary material, further inquiries can be directed to the corresponding author.
